# Synthesis of MAX Phase Nanofibers and Nanoflakes and the Resulting MXenes

**DOI:** 10.1002/advs.202205509

**Published:** 2022-11-18

**Authors:** Hui Shao, Sha Luo, Armel Descamps‐Mandine, Kangkang Ge, Zifeng Lin, Pierre‐Louis Taberna, Yury Gogotsi, Patrice Simon

**Affiliations:** ^1^ Materials Science Department‐CIRIMAT Université Paul Sabatier Toulouse 31062 France; ^2^ Réseau sur le Stockage Electrochimique de l'Energie (RS2E) FR CNRS Amiens 80039 France; ^3^ College of Chemistry and Chemical Engineering Lanzhou University Lanzhou 730000 China; ^4^ Centre de Microcaractérisation Raimond Castaing FR CNRS Toulouse 31400 France; ^5^ College of Materials Science and Engineering Sichuan University Chengdu 610065 China; ^6^ A.J. Drexel Nanomaterials Institute and Department of Materials Science and Engineering Drexel University Philadelphia PA 19104 USA; ^7^ Institut Universitaire de France Paris 75005 France

**Keywords:** layered ternary carbide, MAX phase, molten salt, nanofibers, nanoflakes, nanosized multilayered MXene, pseudocapacitive Li‐ion storage

## Abstract

Layered ternary carbides and nitrides, also known as MAX phases, have attracted enormous attention for many applications, especially as precursors to produce 2D metal carbides and nitrides called MXenes. However, it is still challenging to tune and control the shape/morphology of MAX phase particles at the nanoscale, as they are typically manufactured as large grains using ceramic technology. Herein, nanostructured Ti‐Al‐C MAX phases with fine‐tuned morphology of nanofibers and nanoflakes are prepared by using 1D and 2D carbon precursors at a synthesis temperature of 900 °C. The nanostructured MAX phases are used as precursors to produce nanosized multilayered MXenes, with a considerably shorter etching time and a low reaction temperature. These nanosized MXenes exhibit good electrochemical lithium‐ion storage properties and a pseudocapacitive electrochemical signature. The obtained Ti_2_CT*_x_* MXene electrode can deliver delithiation capacity of 300 mAh g^−1^ at low rates and 100 mAh g^−1^ when the lithiation/delithiation cycle happens within 30 s. Availability of nanoscale MAX phases and MXene nanoflakes with small lateral size may open new opportunities for both classes of materials.

## Graphical abstract

Nanostructured Ti‐Al‐C MAX phases with fine‐tuned morphology of nanofibers and nanoflakes are synthesized by using 1D and 2D carbon precursors. Nanosized multilayered MXenes are produced based on the nanostructured MAX phases, exhibiting good electrochemical lithium‐ion storage properties with pseudocapacitive electrochemical characteristics.

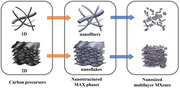

## 1 Introduction

MAX phases are layered ternary carbides and nitrides with the general formula of “M*_n_*_+1_AX*_n_*”, where “M” represents an early transition metal, “A” is an A‐group element, “X” is carbon and/or nitrogen, and *n* is equal to 1, 2, 3, or 4, which indicates the number of M element layers separating the A layers.^[^ ^1^ ^]^ There is a unique combination of properties among the MAX phases, including metal‐like electrical and thermal conductivity, good machinability, thermal shock resistance, and damage tolerance.^[^ ^2^ ^]^ Moreover, they also possess the features of common ceramics, such as high Young's modulus, oxidation resistance, and high‐temperature stability. This unique combination makes MAX phases suitable candidates to bridge the gap between metallic and ceramic materials.^[^ ^2^ ^]^ As such, MAX phases have many applications, including serving as high‐temperature heating elements, structural and coating materials, electrical contacts, radiation‐tolerant materials, etc.^[^ ^1^, ^3^ ^]^

More importantly, MAX phases can be used as precursors for synthesis of 2D metal carbides and nitrides called MXenes. MXenes are obtained by selective removal of the A element from MAX precursors and have the formula M*_n_*_+1_X*_n_*.^[^ ^4^ ^]^ The outstanding properties of MXenes in terms of electrical conductivity, surface area, 2D surface reactivity, etc., make them attractive for a broad range of applications, from energy storage (batteries, supercapacitors)^[^ ^5^ ^]^ and electrocatalysis,^[^ ^6^ ^]^ to medical (photothermal therapy and dialysis),^[^ ^7^ ^]^ communications (electromagnetic shielding and antennas),^[^ ^8^ ^]^ gas separation,^[^ ^9^ ^]^ and water purification.^[^ ^10^ ^]^ The rise of MXenes has boosted the interest in MAX synthesis. Serving as precursors for MXenes is the fastest growing application of MAX phases today. Nontoxic and environmentally friendly titanium‐based MXenes, built of abundant elements, and their hybrids and composites with polymers, ceramics, and metals, are particularly attracting attention.^[^ ^1b^ ^]^

Great efforts have been concentrated on producing new MAX phases, and more than 155 MAX compositions were reported to date and more to count.^[^ ^11^ ^]^ However, while the MAX phase composition can be tuned by selecting the right ratio of precursors, it is still challenging to control the shape and morphology of the MAX particles at the nanoscale. This lack of fine‐tuned nanostructured MAX phase is partially due to the synthesis conditions, since the formation of a high‐purity MAX phase generally requires high temperature, sometimes even high pressure.^[^ ^1a^ ^]^ Taking well‐known Ti‐Al‐C MAX phases as an example, a high temperature of 1100 °C is needed to form the Ti_2_AlC in a spark plasma sintering method^[^ ^12^ ^]^ while an even higher temperature of 1300 °C is reported in a hot pressing synthesis route.^[^ ^13^ ^]^ In addition to the synthesis conditions, the micrometer‐sized precursors used for the MAX synthesis, such as Ti, Al, graphite, and TiC, resulted in micrometer‐sized MAX phase particles. Recently, nanosized Ti_3_AlC_2_ powders were prepared by using nanosized carbon precursors with a molten salt method, shedding light on the synthesis of the nanostructure MAX phase.^[^ ^14^ ^]^ Hollow microrods have also been reported.^[^ ^15^ ^]^ However, the synthesis of nanostructured MAX phase with finely controlled crystal size and particle morphology has not yet been reported. Indeed, the successful preparation of MAX phases with low dimensionality, for instance, nanofibers or nanoflakes, would change the properties of MAX phases thanks to smaller diffusion distance and electron confinement, higher surface area which would allow the use of MAX phases in polymer matrix composites, porous filtration membranes, electromagnetic interference shielding, and other applications. It would also open the road to the direct preparation of nanosized MXenes from nanostructured MAX precursors, with improved properties.

In this paper, nanostructured Ti‐Al‐C MAX phases with fine‐tuned morphology of nanofibers and nanoflakes were prepared by using the molten salt method. The structure and shape of the nanosized MAX phases were controlled by the carbon precursor used. The selected 1D and 2D carbon precursors played a key role in formation of nanostructured MAX phases with unique morphology. Nanosized multilayered MXenes were further obtained by using these nanostructured MAX phases as precursors in a molten salt etching route with a rather low etching temperature and short etching time. These MXenes exhibited good electrochemical Li‐ion storage properties with typical pseudocapacitive characteristics, a delithiation capacity of around 300 mAh g^−1^ at a low rate was measured for a Ti_2_CT*_x_* MXene electrode, that could still deliver 100 mAh g^−1^ when the lithiation/delithiation process happens at a supercapacitor device charge/discharge rate, within 30 s.

## 2 Results and Discussion

### 2.1 Synthesis of Nanostructured MAX Phase with Tunable Morphology

**Figure 1:**
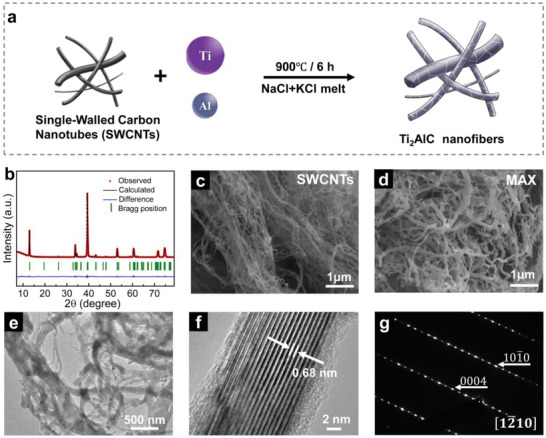
Synthesis of Ti_2_AlC MAX phase nanofibers from 1D SWCNTs carbon precursor. a) Schematic illustration of the synthesis process. b) Rietveld refinement of the XRD pattern of Ti_2_AlC MAX phase nanofibers. SEM images of c) SWCNTs and d) MAX phase and e) TEM images of MAX phase show the morphological evolution from carbon precursors to Ti_2_AlC nanofibers. f) HRTEM image of Ti_2_AlC nanofibers and g) SAED pattern with incident beam parallel to the zone axes of $\left[1\bar{2}10\right]$.

Targeting synthesis of MAX nanofiber powders, single‐walled carbon nanotubes (SWCNTs) with a typical 1D morphology were selected as the carbon precursor. A practically important MAX phase, Ti_2_AlC, was prepared as a proof of concept. As illustrated in **Figure** **1**a, Ti and Al metal powders and SWCNTs were used as precursors for preparing Ti_2_AlC MAX phase nanofibers. The precursors were mixed with a eutectic mixture of NaCl and KCl (0.506:0.494 in molar ratio) and heated up to 900 °C with a dwell time of 6 h under Ar atmosphere (more details in the Experimental Section). Note that the melting point of the NaCl and KCl eutectic mixture is 657 °C.^[^ ^16^ ^]^ The MAX formation reaction was set to take place in a molten salt environment by taking advantage of the enhanced ion diffusion rate in the liquid.^[^ ^1a^ ^]^ Figure 1b gives the X‐ray diffraction (XRD) pattern and Rietveld refinement results of the final powder product. A hexagonal crystal structure (space group of P6_3_/mmc) with the lattice parameters *a* = 0.3054 nm, *c* = 1.3670 nm, and *γ* = 120.0120° was identified, which confirms the formation of Ti_2_AlC MAX phase.^[^ ^17^ ^]^ The average crystallite size of the Ti_2_AlC nanofibers was estimated to be 55 nm by using the Scherrer equation based on the (002) peak. In addition, only one set of Bragg positions was Rietveld refined and matched well with the observed results, indicating the high purity of the final product. More intriguingly, the 1D morphology of the SWCNTs precursor (Figure 1c) was largely preserved in Ti_2_AlC. As evidenced in the scanning electron microscope (SEM) images (Figure 1d and Figure S1, Supporting Information), as well as the transmission electron microscopy (TEM) images Figure 1e, Ti_2_AlC with a typical nanofibrous morphology was obtained. The obtained MAX nanofibers had length in the range of a few to tens of micrometers and widths within a hundred of nanometers, with some as of with as 15 nm, as shown in Figure 1f. This variation in particle sizes may be related to the different degrees of aggregation of SWCNTs. The high‐resolution TEM (HRTEM) image in Figure 1f presents the microstructure of the Ti_2_AlC nanofiber, displaying a typical layered MAX phase with the lattice fringe spacing of 0.68 nm, in agreement with powder XRD data. Selected‐area electron diffraction (SAED) pattern presented in Figure 1g was indexed as $\left[1\bar{2}10\right]$ of the Ti_2_AlC phase.^[^ ^18^ ^]^ Moreover, a homogenous distribution of Ti and Al elements throughout the nanofibers was found from energy‐dispersive spectroscopy (EDS) analysis in SEM (Figure S2, Supporting Information). Using the aforementioned evidence, we demonstrated the successful preparation of Ti_2_AlC MAX phase nanofibers. To the best of our knowledge, this is the first report of MAX phase with such nanofibrous morphology. High‐purity Ti_2_AlC MAX phase with a similar nanofibrous morphology could be also produced at a higher temperature of 1000 °C with a shorter dwell time of 4 h (Figures S3 and S4, Supporting Information).

**Figure 2:**
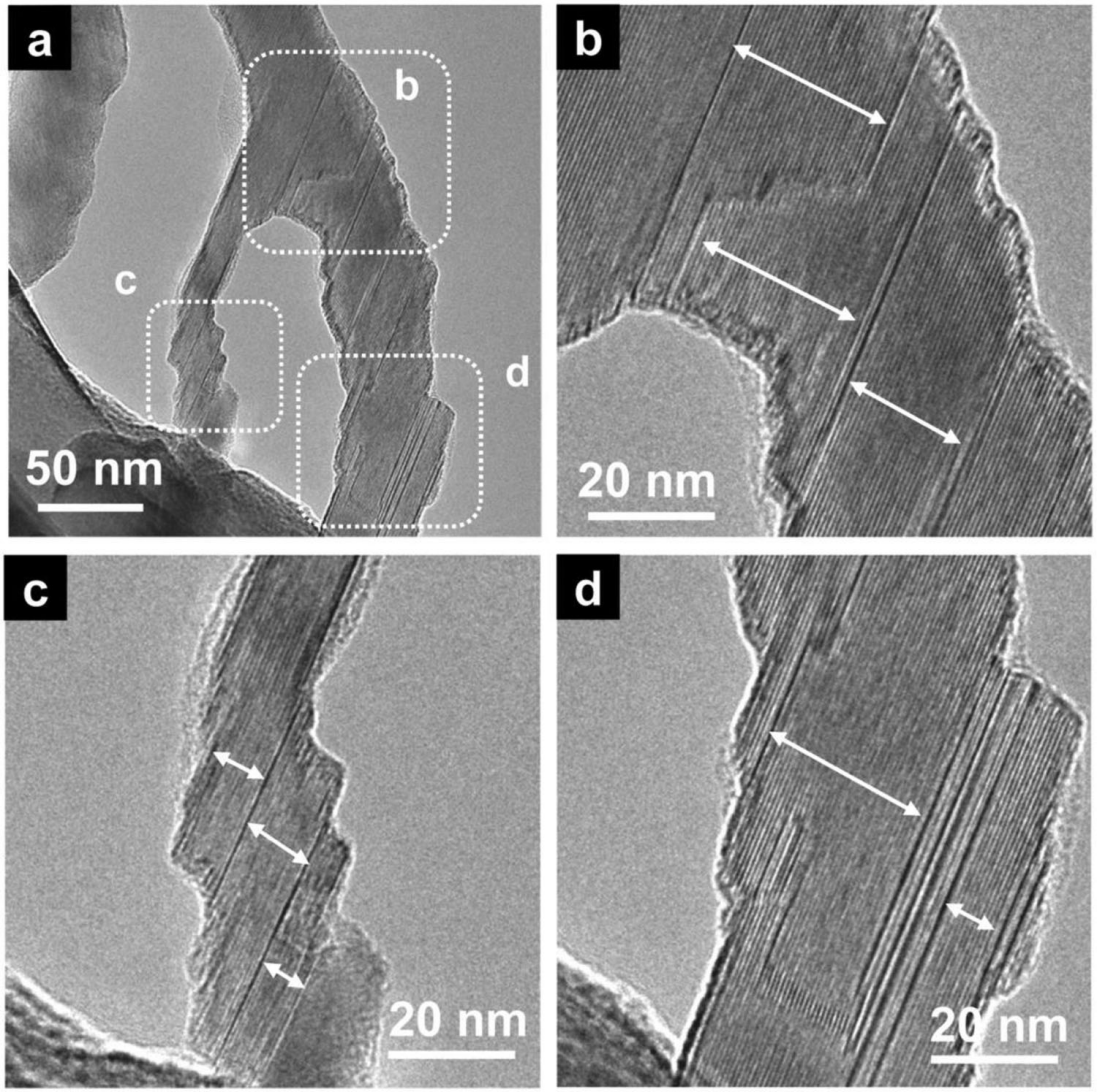
TEM images of MAX phase grains and grain boundaries of the Ti_2_AlC nanofibers. a) TEM image of a typical Ti_2_AlC nanofiber, and the zooming parts labeled inside of (a) are presented in (b)–(d).

TEM analysis in **Figure** **2** shows the microstructure of a single Ti_2_AlC MAX nanofiber. It can be seen that the MAX nanofiber is composed of several grains with clearly visible grain boundaries. However, the crystal orientation remains the same in large segments of the fibers. The stacking faults of MAX grains are also observed, with the absence of any secondary glass phase. Grain boundaries are reported important to the high‐temperature properties of the polycrystalline MAX phase.^[^ ^18^ ^]^ In Ti_2_AlC nanofiber, the secondary‐phase‐free grain boundaries could be beneficial to its high‐temperature oxidation resistance.

**Figure 3:**
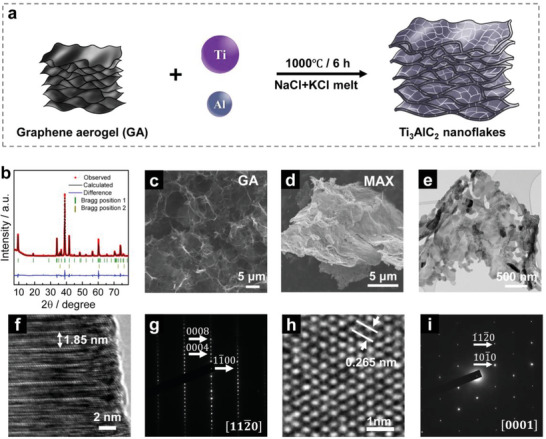
Synthesis of Ti_3_AlC_2_ MAX phase nanoflakes from graphene aerogel precursor. a) Schematic illustration of the synthesis process. b) Rietveld refinement of the XRD pattern of Ti_3_AlC_2_ MAX phase nanoflakes. Morphological evolution from carbon precursors to Ti_3_AlC_2_ nanoflakes is evidenced by SEM images of c) graphene aerogel and d) MAX phase and e) TEM images of MAX phase. f) HRTEM images of Ti_3_AlC_2_ nanoflakes and h) corresponding SAED patterns with incident beam parallel to the g) $\left[11\bar{2}0\right]$ zone axes and i) [0001] zone axes, respectively.

To further validate the assumption that the morphology of the MAX phase is controlled by the morphology of the carbon precursor, the synthesis of MAX phase nanoflake powders was performed by using 2D carbon. Graphene aerogel (GA) was prepared from 2D graphene, which locally retains its 2D morphology, was selected as a carbon source.^[^ ^19^ ^]^ Details of GA preparation are described in the Experimental Section. As explained in the schematic diagram (**Figure** **3**a), the Ti_3_AlC_2_ nanoflakes were prepared using Ti, Al, and GA precursors in a molten salt environment at 1000 °C. The XRD pattern and Rietveld refinement results are presented in Figure 3b. Two sets of Bragg positions were Rietveld refined and showed formation of Ti_3_AlC_2_ with a hexagonal crystal structure (space group of P6_3_/mmc) having the lattice parameters *a* = 0.3068 nm, *c* = 1.8527 nm, and *γ* = 120.0°.^[^ ^17^ ^]^ A small amount of cubic TiC was also found. The synthesized Ti_3_AlC_2_ was 96 wt% pure, according to the Rietveld refinement analysis. The average size of Ti_3_AlC_2_ crystallites in nanoflakes was estimated to be 43 nm by using the Scherrer equation based on the (002) peak. Figure 3c shows the typical morphology of the GA precursor, having a porous network structure consisting of thin wrinkled layers of stacked graphene sheets. After the MAX formation, a thin‐flake morphology was observed for the Ti_3_AlC_2_, as shown in SEM (Figure 3d and Figure S5, Supporting Information) and TEM (Figure 3e) images. In addition, the MAX nanoflakes show wrinkles, like the graphene aerogel precursor. This further indicates that the morphology of the MAX phase is highly dependent on the carbon precursors. The length and width of these MAX flakes range from a few micrometers to tens of micrometers, with a thickness within a hundred of nanometers. The particle size of the MAX phase nanoflakes is highly dependent on the size of graphene flakes. Typically, a MAX nanoflake is composed of grains with different orientations, as illustrated in Figure S6a (Supporting Information). The grain boundaries can also be observed in the SEM images in Figure S6b (Supporting Information). HRTEM images in Figure 3f,h present the microstructure of Ti_3_AlC_2_ nanoflakes, showing a stacking sequence with a periodicity of 1.85 nm along with the [0001] direction and a regular hexagonal arrangement of the atoms, respectively. The corresponding SAED patterns are shown in Figure 3g,i, which can be indexed as $\left[11\bar{2}0\right]$ and [0001] of the Ti_3_AlC_2_ phase, respectively. SEM‐EDS mapping images in Figure S7 (Supporting Information) show uniformly distributed Ti and Al elements in the Ti_3_AlC_2_ nanoflakes. Moreover, as demonstrated in Figures S8–S11 (Supporting Information), Ti_2_AlC nanoflakes with a purity of 98 wt% (Rietveld refinement analysis of XRD results) were also synthesized with a similar procedure at 900 °C by adjusting the ratio of starting precursors (details are summarized in Table S1, Supporting Information). This is the first report on the preparation of MAX phase nanoflake powders.

The successful preparation of nanostructured MAX phase materials with controlled morphology is mainly attributed to the molten salt system and the carbon precursors. It was reported that liquid medium enables rapid ion diffusion and thus facilitates the MAX phase formation.^[^ ^20^ ^]^ However, micrometer‐sized graphite was often used as the carbon source in the previous reports, requiring temperatures well over 1000 °C for forming MAX phases. Taking Ti‐Al‐C ternary carbides as an example, reaction temperatures of over 1000 and 1250 °C were reported for the synthesis of high‐purity Ti_2_AlC and Ti_3_AlC_2_ MAX phases, respectively.^[^ ^20a,c^ ^]^ In comparison, the synthesis temperatures of high‐purity Ti_2_AlC and Ti_3_AlC_2_ in this work are 900 and 1000 °C, respectively, which indicates that the nanosized carbon precursors promote the MAX phase formation reaction.^[^ ^14^ ^]^ Also, the MAX phase products prepared from micrometer‐scale graphite usually have particle sizes larger than tens of micrometers with uncontrollable crystal size and morphology, requiring crushing and milling before use.^[^ ^1a^ ^]^ The morphology of Ti‐Al‐C MAX phases is largely determined by the carbon precursor, since a template‐mediated growth mechanism takes place in the MAX formation.^[^ ^21^ ^]^ Therefore, 1D and 2D carbon precursors lead to formation of MAX nanofibers and nanoflakes, respectively. We anticipate that this method can be generalized to produce nanostructured MAX phases with tunable morphology beyond Ti‐Al‐C carbides, since there are more than 155 currently known MAX phase compositions and majority of them are carbides.^[^ ^11^ ^]^

To further understand the reaction path leading to formation of these nanostructured MAX phases, two sets of experiments using graphene aerogel precursors with starting materials ratios targeting Ti_2_AlC and Ti_3_AlC_2_ MAX phases were annealed, respectively, at 900 and 1000 °C for 10 min. The intermediate compounds during the MAX formation were identified by XRD and shown in Figure S12 (Supporting Information). After being held at 900 °C for 10 min, a large amount of TiC and TiAl intermetallic and some Ti_2_AlC phases formed. Previous work using graphite as the carbon source suggested that TiC formed on the graphite, and a TiAl intermetallic formed on the titanium surface when titanium was surrounded with melted aluminum at elevated temperatures.^[^ ^20a^ ^]^ Following this postulate, we proposed that in our case TiC formed when graphene came in contact with titanium and it rapidly reacted with TiAl intermetallic to form Ti_2_AlC grains. These reactions occurred simultaneously in multiple sites of the wrinkled and stacked graphene flakes, forming Ti_2_AlC grains with multiple orientations and therefore maintaining the flake morphology. The Ti_2_AlC grain growth stopped once the TiC was depleted locally. This reaction pathway offers a reasonable explanation of the formation of Ti_2_AlC nanoflakes, and a similar reaction process can be proposed for the formation of Ti_2_AlC nanofibers. In the case of Ti_3_AlC_2_ nanoflakes, Ti_2_AlC, TiC and the final product, Ti_3_AlC_2_, were found by XRD after the reactants were heated up to 1000 °C with a short dwell time of 10 min (Figure S12b, Supporting Information). It is proposed that the formed Ti_2_AlC grains further react with TiC to form the final product Ti_3_AlC_2_, in agreement with previous literature.^[^ ^22^ ^]^

### 2.2 MXenes Derived from Nanostructured MAX Phases

**Figure 4:**
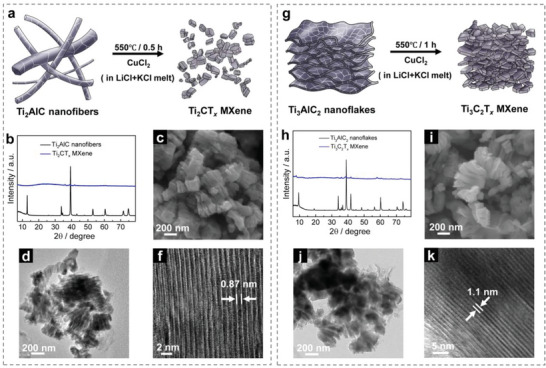
MXenes derived from nanostructured MAX phase precursors. Schematic illustrations of Ti_2_CT*_x_* MXene prepared from a) Ti_2_AlC nanofibers and g) Ti_3_C_2_T*_x_* MXene prepared from Ti_3_AlC_2_ nanoflakes. XRD patterns of MXenes and corresponding pristine MAX phases: b) Ti_2_CT*_x_* MXene prepared from Ti_2_AlC nanofibers; h) Ti_3_C_2_T*_x_* MXene prepared from Ti_3_AlC_2_ nanoflakes. SEM images of c) Ti_2_CT*_x_* MXene and i) Ti_3_C_2_T*_x_* MXene showing the particle morphology and TEM images of d) Ti_2_CT*_x_* MXene and j) Ti_3_C_2_T*_x_* MXene. HRTEM images of f) Ti_2_CT*_x_*‐NI and k) Ti_3_C_2_T*_x_* MXene show the lattice fringes of obtained MXenes, confirming the nanolaminate nature of the final products.

One of the most important applications of MAX phases is their use as precursors for producing MXenes. The aforementioned nanostructured Ti‐Al‐C MAX phases, including Ti_2_AlC nanofibers, Ti_2_AlC nanoflakes, and Ti_3_AlC_2_ nanoflakes, were used to prepare MXenes by etching in molten salt.^[^ ^23^ ^]^ As illustrated in schematic **Figure** **4**a,g, pristine MAX phase powders were immersed in a Lewis acid melt with a eutectic mix of LiCl and KCl (0.592:0.408 in molar ratio, melting point of 353 °C). During the synthesis, aluminum is selectively removed from the MAX by reaction with CuCl_2_, resulting in a mixture of MXene and Cu metal.^[^ ^23^ ^]^ Figure S13 (Supporting Information) gives the typical XRD pattern after the etching reaction, showing prominent sharp peaks belonging to metallic copper, with the absence of peaks of the MAX phase precursor, indicating the complete etching of the pristine MAX phase. A low‐intensity peak located at 2*θ* of 10.21° (corresponding to an interlayer distance of 0.87 nm) was also recorded, which matches well with the (002) peak of Ti_2_CT*_x_* MXene.^[^ ^24^ ^]^ The MXene/Cu mixtures were further treated with 0.2 m FeCl_3_ solution to remove the copper. XRD patterns of final MXene products after the removal of Cu as well as their corresponding pristine MAX phases are presented in Figure 4b,h and Figure S14 (Supporting Information). In the XRD pattern of the Ti_3_C_2_T*_x_* MXene (Figure 4h), the (002) peak of the Ti_3_AlC_2_ MAX phase is shifted from 2*θ* angle of 9.58° to 8.05°, which corresponds to an expansion of interlayer distance from 0.926 to 1.1 nm, in agreement with Ti_3_C_2_T*_x_* MXene prepared from the molten salt method.^[^ ^23^, ^25^ ^]^ For Ti_2_CT*_x_* MXenes prepared from Ti_2_AlC nanofibers and nanoflakes, the (002) peak disappears after FeCl_3_ treatment. This is similar to the previous observation of molten salt method derived Ti_2_CT*_x_* MXene,^[^ ^25b^ ^]^ and is attributed to more random arrangement of Ti_2_C layers compared to Ti_3_C_2_ layers.

SEM images of Figure 4c,i and Figures S15–S17 (Supporting Information) show morphology of the obtained MXenes. It can be seen that the features of parent MAX phases—nanofibers and nanoflakes—disappeared, while an accordion‐like structure was observed for all the products, indicating the successful preparations of 2D MXenes, in agreement with the previous report.^[^ ^4b^ ^]^ Interestingly, after the etching reaction, the MAX phase secondary particles composed of many MAX grains were broken down to small MXene particles with open structures. Similar morphology evolution from MAX phases to MXenes was also evidenced by TEM analysis, as shown in Figure 4d,j. The width of most MXene particles was less than 500 nm, due to the nanosized MAX phase precursors. HRTEM images show the microstructures of Ti_2_CT*_x_* prepared from Ti_2_AlC nanofibers (Figure 4f) and Ti_3_C_2_T*_x_* derived from Ti_3_AlC_2_ nanoflakes (Figure 4k), where lamellar microstructures with the spacings of 0.87 and 1.1 nm were observed for Ti_2_CT*_x_* MXene and Ti_3_C_2_T*_x_* MXene, respectively.

It usually took either a long time (a few hours to 24 h) or a high temperature (650–700 °C) to complete the etching reaction of the MAX phase into MXene when micrometer‐sized MAX phases in molten salt were used as precursors.^[^ ^23^, ^25^, ^26^ ^]^ By taking advantage of the nanosized MAX precursors, short reaction times of 0.5 and 1 h at the low etching temperature of 550 °C were found to be enough for producing Ti_2_CT*_x_* and Ti_3_C_2_T*_x_* MXene, respectively, thanks to the short ion diffusion path during the etching reactions.

The nanosized multilayered Ti_2_CT*_x_* prepared from Ti_2_AlC nanofibers (noted as Ti_2_CT*_x_*‐NI) and Ti_2_AlC nanoflakes (noted as Ti_2_CT*_x_*‐NA) and Ti_3_C_2_T*_x_* nanoflakes MXene prepared from Ti_3_AlC_2_ nanoflakes were further investigated as negative electrodes for Li‐ion intercalation in LiPF_6_‐based electrolyte. The initial cyclic voltammetry (CV) profiles of MXene electrodes at a low scan rate of 0.5 mV s^−1^ are presented in Figure S18 (Supporting Information). An irreversible capacity was observed for all three MXene electrodes during the first lithiation, which was related to the formation of solid electrolyte interphase (SEI).^[^ ^23^, ^25^ ^]^ CV tests were further performed at various potential scan rates from 0.5 to 100 mV s^−1^. Figure S19a (Supporting Information) shows the CV profiles of Ti_2_CT*_x_*‐NI MXene electrode at the scan rate of 1 and 5 mV s^−1^, showing a symmetrical CV profile with the absence of any visible redox peaks during the Li intercalation/deintercalation process. This suggests a fast, pseudocapacitive Li‐ion storage, in agreement with the electrochemical signatures of the previous molten salt derived MXenes.^[^ ^23^, ^25^, ^27^ ^]^ In addition, the surface reactions dominate the Li‐ion intercalation/deintercalation kinetics according to the electrochemical impedance spectroscopy and power law analysis (Figure S19, Supporting Information). Similar electrochemical signatures were observed for Ti_3_C_2_T*_x_* MXene electrodes and Ti_2_CT*_x_*‐NA (Figures S20c and S21a, Supporting Information). The specific capacities were calculated based on the anodic scans which related to the delithiation process and presented in Figure S21b (Supporting Information). At a low scan rate of 0.5 mV s^−1^ (corresponds to 96.7 min), a specific capacity of 302 mAh g^−1^ was measured for the Ti_2_CT*_x_*‐NI electrode. For Ti_3_C_2_T*_x_* electrode, a lower specific capacity of 188 mAh g^−1^ was obtained. When the scan rate increases to 100 mV s^−1^, corresponding to a complete lithiation/delithiation in 29 s, specific capacities of 104 mAh g^−1^ and 74 mAh g^−1^ were delivered for Ti_2_CT*_x_*‐NI electrode and Ti_3_C_2_T*_x_* electrode, respectively, highlighting the high‐rate capability of the nanosized MXene electrodes. The galvanostatic charge–discharge (Figure S20c, Supporting Information) of the Ti_2_CT*_x_*‐NI electrode shows a sloping profile without any plateau, as a result of the pseudocapacitive charge storage mechanism. A specific capacity of about 315 mAh g^−1^ was found at a specific current of 0.2 A g^−1^ for the Ti_2_CT*_x_*‐NI electrode. It can still deliver about 96 mAh g^−1^ when increasing the charge/discharge current to 20 A g^−1^, highlighting again the high‐rate electrochemical performance of the Ti_2_CT*_x_*‐NI electrode. These results from galvanostatic tests were in good agreement with the CV tests. Similar results were also obtained for Ti_2_CT*_x_* electrodes obtained from the Ti_2_AlC nanoflakes (Figure S21 and Table S4, Supporting Information), showing the remarkable electrochemical properties of these nanosized multilayered MXenes, which can operate at the rates typical for electric double‐layer capacitors, but store significantly more energy.

## 3 Conclusion

Nanostructured Ti‐Al‐C MAX phase powders with tunable nanofiber and nanoflake morphologies were prepared using a molten salt synthesis method. This unique morphology of MAX phase nanostructures was achieved due to the use of 1D (nanotubes) and 2D (graphene) carbon precursors. The availability of fibrous and platelet morphologies should expand the range of MAX phase applications to thin films, composites, etc. The reaction path of nanostructured MAX phases shown in this paper could be potentially extended beyond the Ti‐Al‐C system to a large number of compositions, depending on the desired properties. Nanosized multilayered MXenes were successfully prepared from these MAX phases, with a considerably shorter etching time and a lower reaction temperature. These nanosized multilayered MXenes exhibited good electrochemical lithium‐ion storage properties, demonstrating a typical pseudocapacitive electrochemical signature with 50% capacity increase and excellent high‐rate capabilities. More specifically, the Ti_2_CT*_x_* electrode can deliver a delithiation capacity of around 300 mAh g^−1^ at low rates and over 100 mAh g^−1^ when the lithiation/delithiation process happens within 30 s, reaching the time domain of supercapacitors, but still using Li ion intercalation/deintercalation reactions in organic electrolyte, which lead to a much larger energy stored. Although we highlight here the energy storage application of the nanosized MXenes, their use in electrocatalysis, gas separation, multifunctional composites and biomedical applications may be equally beneficial.

## 4 Experimental Section

### Synthesis of MAX Phase Nanofibers

Ti_2_AlC nanofibers were prepared using Ti metal powder (purity of 99.99%, ≈325 mesh, Alfa Aesar), Al metal powder (purity of 99.5%, ≈325 mesh, Alfa Aesar), and SWCNT (content ≈75 wt%, TUBALL) as starting materials. SWCNTs were premixed with an eutectic mixture of NaCl and KCl (NaCl:KCl = 0.506:0.494 in molar ratio, noted as NaCl+KCl, purity ≥ 99% for both salt, Alfa Aesar) in a 1:10 weight ratio, immersed in deionized water, and then probe‐sonicated to form a suspension. The suspension was then frozen by liquid nitrogen and freeze‐dried to obtain a SWCNT/salt mixture. Typically, SWCNT/salt mixture (with 0.05 g of SWCNT) was mixed with NaCl+KCl salt and milled for 40 min with mortar and pestle in the presence of ethanol (purity ≥ 99.5%, Sigma‐Aldrich). Afterward, Ti powder was added to the mixture and ground for 10 min. Then Al powder was added to the mixture and ground for another 10 min. The weight ratio of SWCNT:NaCl+KCl salt was 1:30. The molar ratio of Ti:Al:SWCNT was 2:1.2:1. The mixture was dried at 80 °C under vacuum for 1 h and then placed in an alumina crucible and covered with 3 g of NaCl+KCl salt. The crucible with a lid on top was placed inside of a tube furnace and heated to 900 °C with a dwell time of 6 h or to 1000 °C with a dwell time of 4 h under Ar atmosphere. The heating rate was 10 °C min^−1^. A pair of alumina foam blocks was placed on the top and under the bottom of the crucible in the annealing process. After naturally cooling down to room temperature, the mixture was first washed with DI water to remove residual salts and then stirred in 50 mL of 6 m HCl for 6 h to remove any possible intermetallic compounds. Finally, the product was vacuum filtered and washed with deionized water three times to remove residual acid.

### Synthesis of MAX Phase Nanoflakes

Lab‐made graphene aerogel (GA) was used as carbon precursor for the synthesis of MAX phase nanoflakes. The preparation of GA is detailed as follows. Graphene oxide (GO) was first prepared by a modified Hummers method.^[^ ^28^ ^]^ Then reduced graphene oxide (rGO) hydrogel was made from the GO aqueous dispersion with a concentration of 2 mg mL^−1^ by hydrothermal reduction process. Typically, 80 mL of GO aqueous dispersion was sealed in a hydrothermal kettle and heat it up to 160 °C for 6 h. The obtained rGO hydrogel was frozen by liquid nitrogen and freeze‐dried to produce rGO aerogel. Afterward, rGO aerogel was put inside a tube furnace and heated up to 900 °C (heating rate of 10 °C min^−1^) and held for 1 h under Ar atmosphere to further remove the oxygen groups of rGO. The rest procedures for preparing Ti_3_AlC_2_ and Ti_2_AlC nanoflakes were similar to that of Ti_2_AlC nanofibers, except for the carbon sources, raw material ratio and annealing treatment. Details are summarized in Table S1 (Supporting Information).

### Preparation of MXenes from Nanostructured MAX Phases

All MXenes were prepared by using the Lewis acid molten salt route, as summarized in Table S2 (Supporting Information). Taking Ti_2_CT*_x_* MXene derived from Ti_2_AlC nanofibers as an example: 1.2 g of CuCl_2_ (99%, Acros Organics) was mixed with 3 g of eutectic mixture of LiCl (purity ≥ 99%, Alfa Aesar) and KCl (0.592:0.408 in molar ratio) and ground for 10 min, then 0.3 g of Ti_2_AlC MAX phase nanofibers was added to the mixture and ground for another 10 min before transferring to an alumina crucible. The alumina crucible with a lid on top was first heated to 200 °C with a dwell time of 1 h and then up to 550 °C with a dwell time of 0.5 h in a quartz tube under Ar. After naturally cooling down to room temperature, the mixture was washed with DI water to remove residual salts, leaving a MXene/Cu mixture. Afterward, the MXene/Cu mix was stirred in 70 mL of 0.2 m FeCl_3_ solution for 0.5 h to remove Cu. Then, the MXene product was vacuum filtered and washed with deionized water and ethanol for three times. Finally, the MXene product was dried overnight at 80 °C under vacuum.

### Materials Characterization

X‐ray diffraction patterns were recorded in a 2*θ* range of 5°–80° by using a D‐8 diffractometer (Endeavor, Bruker) with Cu‐K*α* radiation (40 kV, 25 mA). A step size of 0.01° and collection time of 1 s step^−1^ was used to collect XRD data for further Rietveld refinement, including Figures 1b and 2b, Figures S3a and S8a (Supporting Information). Other XRD patterns were recorded with a step size of 0.016° and collection time of 0.13 s step^−1^. The microstructure and chemical composition were analyzed by scanning electron microscopy (SEM‐FEG, JSM‐7900F) at 5 kV with EDS (Bruker XFLASH 6‐60) at 15 kV and TEM (JEM‐2100F) at 200 kV.

### Electrochemical Characterization

All electrochemical tests were conducted in a two‐electrode coin cell (CR2032) configuration. MXenes synthesized from various pristine MAX phases were used as active materials. For preparing the working electrodes, MXene powders, acetylene carbon black, and polytetrafluoroethylene (PTFE) were mixed in a weight ratio of 80:12:08 in ethanol to form a film. Then the film was casted onto a Cu foil with presence of ethanol. After drying overnight at 80 °C under vacuum, 12 mm‐diameter discs were cut and used as the working electrodes. The loading mass of MXene electrodes was in range of 1–1.5 mg cm^−2^, based on active materials, and the thickness of MXenes electrodes was between 15 and 23 µm (measured by a digital micrometer, QuantuMike IP65). Li foil (Sigma‐Aldrich, purity of 99.9%, thickness of 0.75 mm, diameter of 13 mm) was used as the counter and reference electrode. One layer of 17 mm‐diameter glass microfiber A (Whatman) was used as a separator, and 1 m LiPF_6_/ethylene carbonate‐dimethyl carbonate (1:1 vol%, Solvionic, purity of 99.9%, water content less than 20 ppm) was used as electrolyte. All cells were assembled in an argon‐filled glovebox, with H_2_O and O_2_ content less than 0.1 ppm.

Electrochemical tests were conducted using a Biologic VMP3 potentiostat and a Lanhe M340A battery tester at room temperature 23 ± 3 °C. Electrochemical impedance spectroscopy (EIS) measurements were carried out at various bias potentials versus Li metal in a frequency range of 10 mHz to 200 kHz (10 points dec^−1^) with a potential amplitude of 10 mV. Before each EIS measurement, linear sweep voltammetry with a scan rate of 0.5 mV s^−1^ was applied to reach the desired potential, followed by a rest time of 10 min at this potential to reach equilibrium. Specific capacity values derived from the cyclic voltammetry profiles were calculated from anodic scan curves following the equation

(1) $$Q_{m}=\frac{\int_{0}^{t}\left|i\right|\;dt}{3.6\;m}$$

where *Q*_m_ (mAh g^−1^) is the gravimetric capacity, *t* is the recording time (s), *i* is the response current (A), and *m* is the mass of the working electrode (g).

## Conflict of Interest

The authors declare no conflict of interest.

## Author Contributions

H.S. and S.L. contributed equally to this work. P.S., Y.G., P.L.T., and H.S. designed the research. H.S. and S.L. conducted material preparations and characterizations. A.D.M. carried out TEM measurement. Z.F.L. performed XRD Rietveld refinement. H.S. conducted the electrochemical test. P.S., Y.G., P.L.T., S.L., and H.S. prepared the manuscript. All authors contributed to the discussion of the data and editing of the manuscript under supervision from P.L.T., Y.G., and P.S.

## Supporting information

Supporting Information

## Data Availability

The data that support the findings of this study are available from the corresponding author upon reasonable request.
