# Supplementary material for: Synthesis of MAX Phase Nanofibers and Nanoflakes and the Resulting MXenes
Source: Adv Sci (Weinh). 2022 Nov 18;10(1):2205509. doi: 10.1002/advs.202205509 (PMC9811477; doi:10.1002/advs.202205509)
Supplement: Supplementary file 1 — Supporting Information [file ADVS-10-2205509-s001.pdf]

Supporting Information for

Synthesis of MAX phase nanofibers and nanoflakes and the resulting MXenes

Hui Shao[1,2,†,*], Sha Luo[1,3,†], Armel Descamps-Mandine[4], Kangkang Ge[1,2], Zifeng Lin[5], Pierre-Louis Taberna[1,2,*], Yury Gogotsi[6,*], Patrice Simon[1,2,7,*]

[1] 1Materials Science Department-CIRIMAT, Université Paul Sabatier, Toulouse, 31062, France

[2] Réseau sur le Stockage Electrochimique de l'Energie (RS2E), FR CNRS, Amiens, 80039, France

[3] College of Chemistry & Chemical engineering, Lanzhou University, Lanzhou, 730000, China

[4] Centre de Microcaractérisation Raimond Castaing, FR CNRS, Toulouse, 31400, France

[5] College of Materials Science and Engineering, Sichuan University, Chengdu, 610065, China

[6] A.J. Drexel Nanomaterials Institute and Department of Materials Science and Engineering, Drexel University, Philadelphia, 19104, USA

[7] Institut Universitaire de France, Paris, 75005, France

*Correspondence to:

Dr. Hui Shao, E-mail: hui.shao@univ-tlse3.fr

Dr. Pierre-Louis Taberna, E-mail: pierre-louis.taberna@univ-tlse3.fr

Prof. Yury Gogotsi, E-mail: gogotsi@drexel.edu

Prof. Patrice Simon, E-mail: patrice.simon@univ-tlse3.fr

† These authors contributed equally to this work.

Table S1. Preparation of MAX phase nanofibers and nanoflakes from single-walled carbon nanotubes (SWCNTs) and lab-made graphene aerogel (GA).

MAX Phase	Carbon precursor	Ratio of starting materials (by mol)	T (°C)	Dwell time (h)
Ti_2AlC nanofibers	SWCNT	Ti:Al:C = 2:1.2:1	900	6
Ti_2AlC nanofibers	SWCNT	Ti:Al:C = 2:1.2:1	1000	4
Ti_2AlC nanoflakes	GA	Ti:Al:C = 2.3:1.3:1	900	6
Ti_3AlC_2 nanoflakes	GA	Ti:Al:C = 3:1.2:2	1000	6

Table S2. MXenes derived from nanostructured MAX phases. Eutectic salt used here is LiCl and KCl (0.592:0.408 in molar ratio).

MXene	MAX precursor	Ratio of starting materials (by weight)	T (°C)	Dwell time (h)
Ti_2CT_x	Ti_2AlC nanofibers	MAX:$CuCl_2$:Salt = 0.3:1.2:3	550	0.5
Ti_2CT_x	Ti_2AlC nanoflakes	MAX:$CuCl_2$:Salt = 0.3:1.2:3	550	0.5
$Ti_3C_2T_x$	Ti_3AlC_2 nanoflakes	MAX:$CuCl_2$:Salt = 0.2:0.55:2	550	1

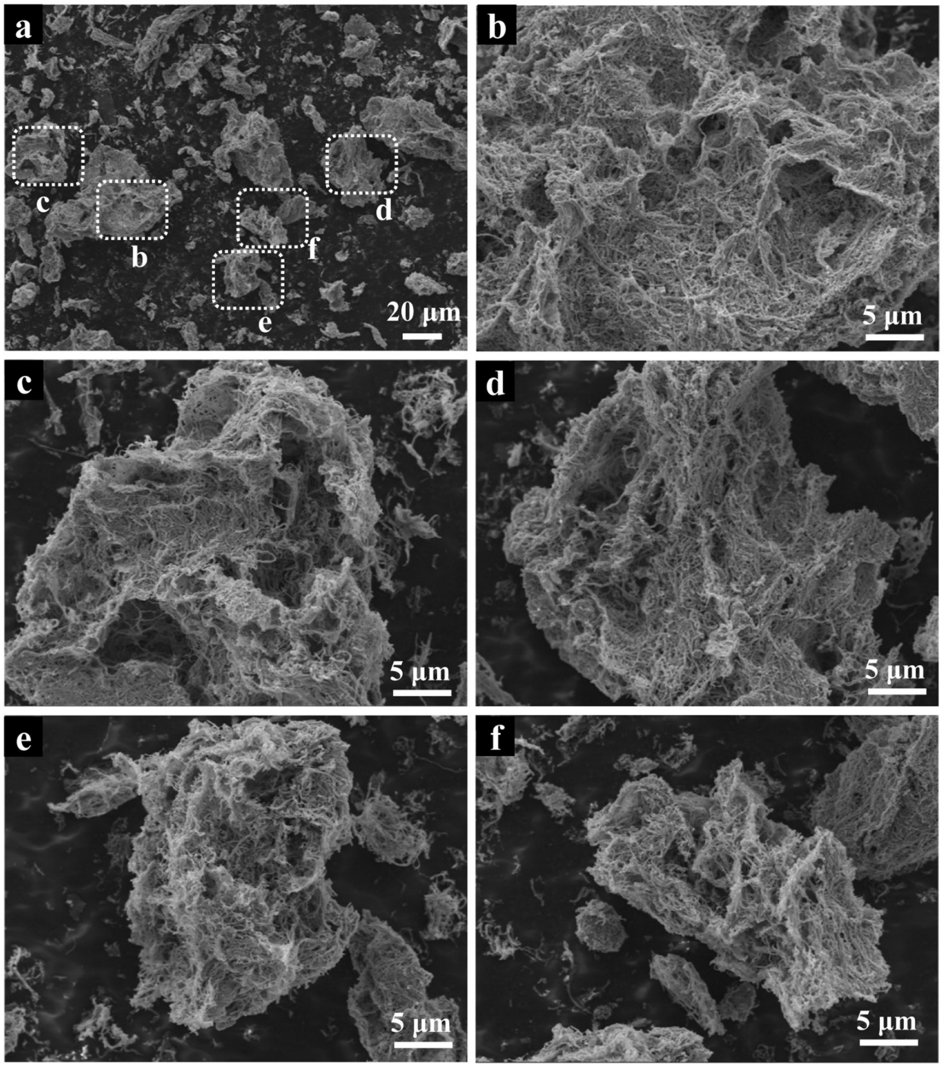

Figure S1. SEM images of the Ti_2AlC MAX phase prepared from 1D carbon precursors at 900 ºC, which show the typical nanofibrous morphology.

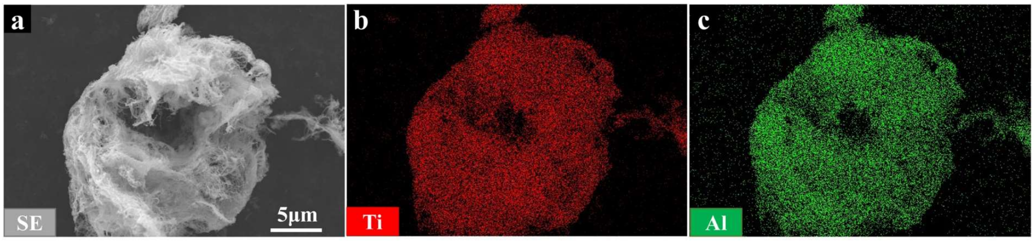

Figure S2. Distribution of Ti and Al in Ti_2AlC nanofibers prepared at 900 ºC. The carbon distribution is not shown due the background noise of carbon tape used in the SEM analysis.

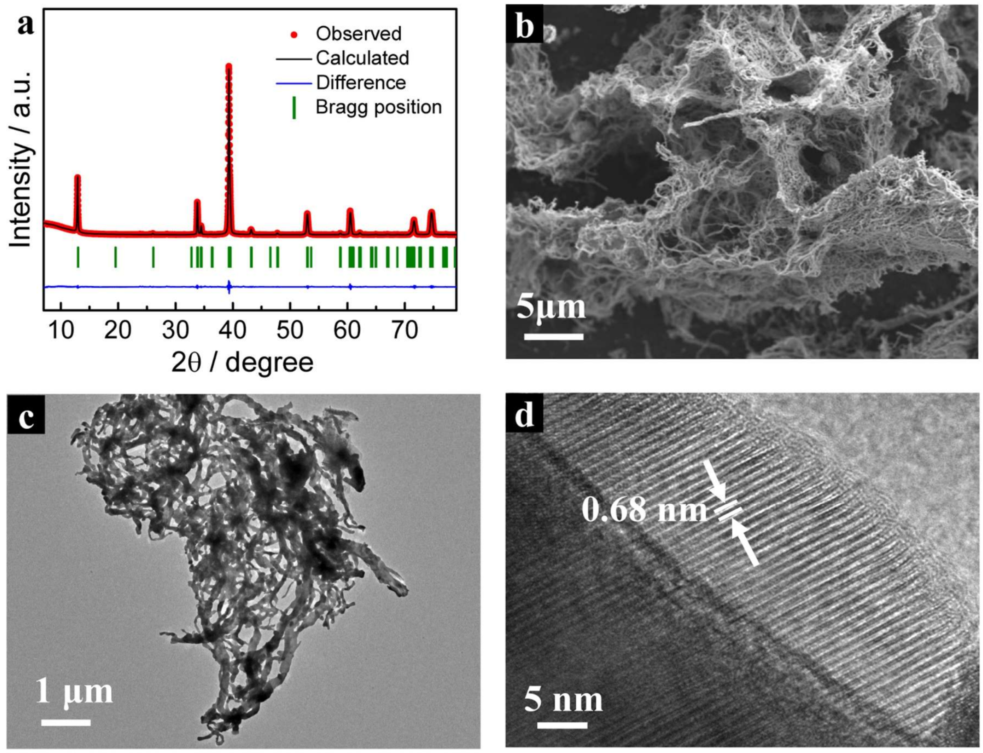

Figure S3. Characterizations of Ti_2AlC nanofibers prepared at 1000 °C. a, Rietveld refinement of the XRD pattern of Ti_2AlC nanofibers. A hexagonal crystal structure (space group of $P6_3/mmc$) with the lattice parameters a=0.3055 nm, c=1.3664 nm and γ=119.9858° (calculated from Rietveld refinements) was identified. SEM image (b) and TEM image (c) show the typical nanofibrous morphology of the obtained MAX phase.

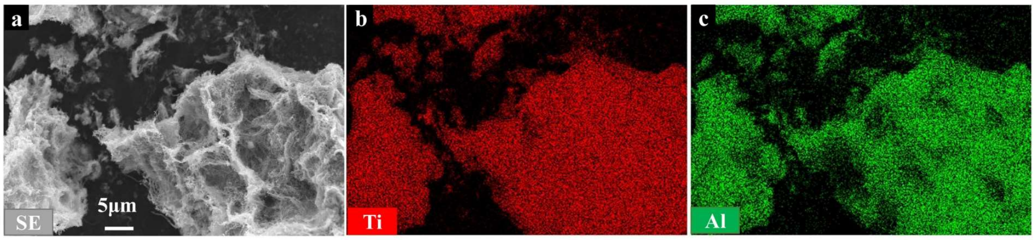

Figure S4. Distribution of Ti and Al in Ti_2AlC nanofibers prepared at 1000 °C. The carbon distribution is not shown due the background noise of carbon tape used in the SEM measurement.

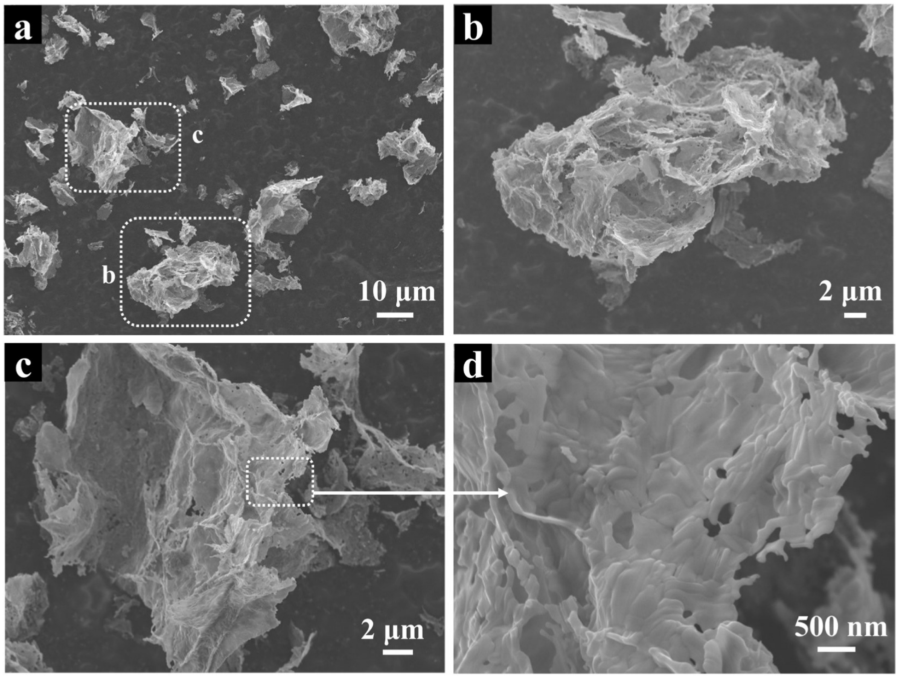

Figure S5. SEM images of the Ti_3AlC_2 MAX phase prepared from the graphene aerogel precursor with various magnifications.

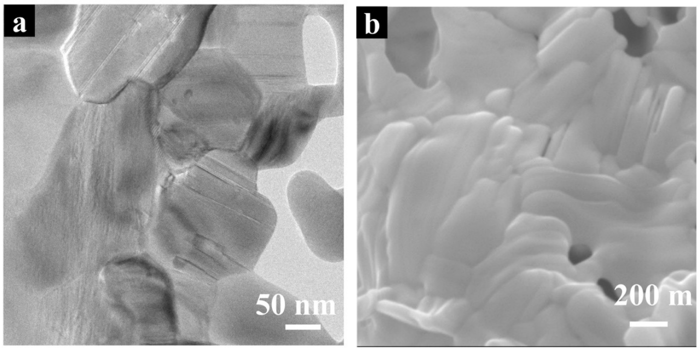

Figure S6. TEM image (a) and SEM image (b) show the MAX phase grains and grain boundaries between the Ti_3AlC_2 nanoflakes.

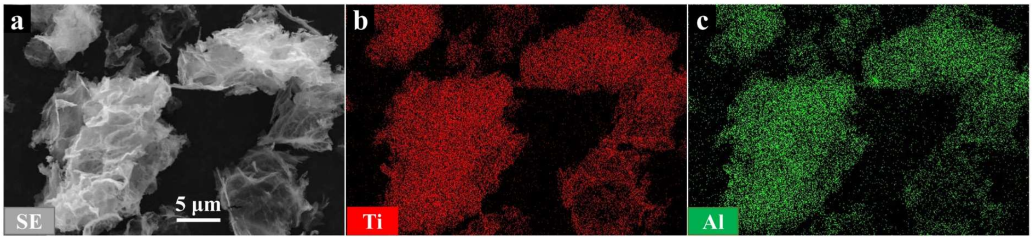

Fig. S7 Distribution of Ti and Al elements in Ti_3AlC_2 nanoflakes. The carbon distribution is not shown due the background noise of carbon tape used in the SEM measurement.

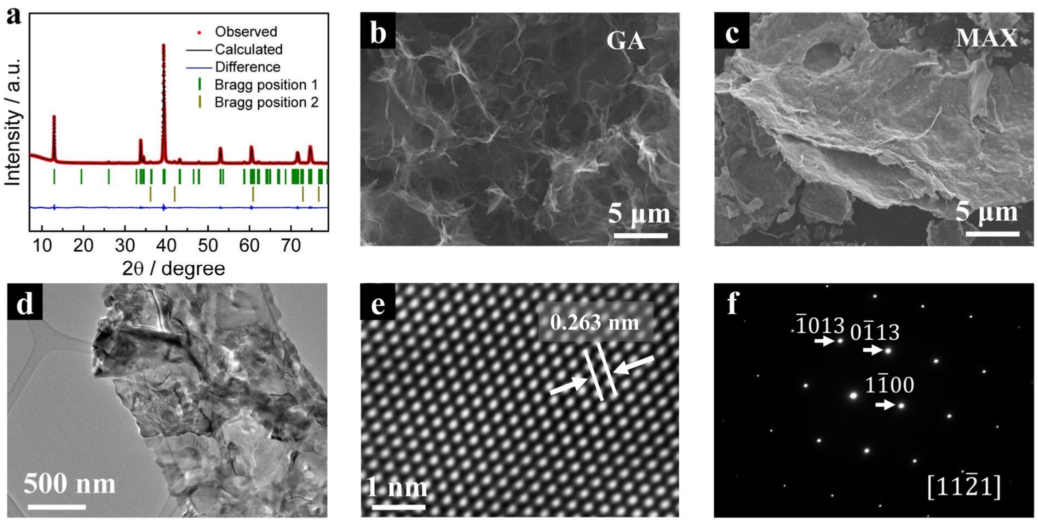

Figure S8. Synthesis of Ti_2AlC MAX phase nanoflakes from graphene aerogel precursor. a, Rietveld refinement of the XRD pattern of Ti_2AlC MAX phase. A hexagonal crystal structure (space group of $P6_3/mmc$) with the lattice parameters a=0.3049 nm, c=1.3655 nm and γ=119.6987° (from Rietveld refinements) with purity of 98.3 wt% was identified. Morphological evolution from carbon precursors to Ti_2AlC nanoflakes was recorded from SEM images of GA (**b**) and MAX phase (**c**) and TEM image of MAX phase (**d**). HR-TEM images of Ti_2AlC nanoflakes (**e**) and corresponding SAED patterns with the incident beam parallel to the $[11\bar{2}1]$ zone axis.

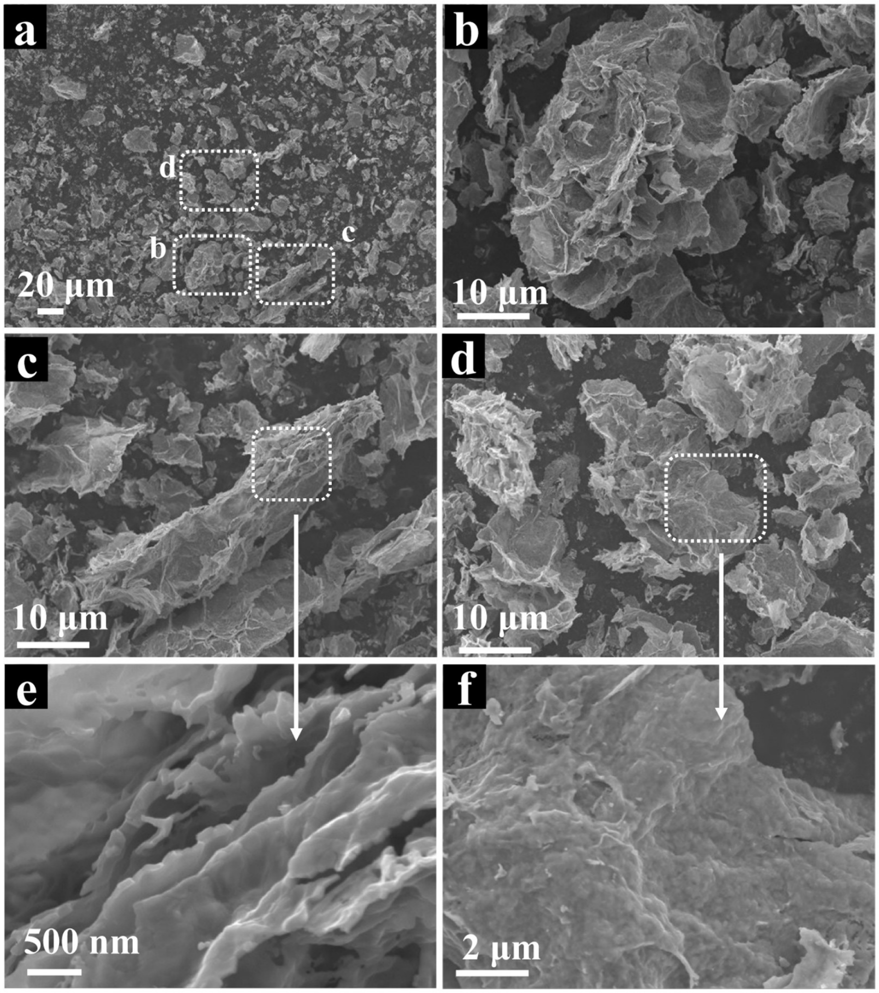

Figure S9. SEM images of the Ti_2AlC MAX phase prepared from the graphene aerogel precursor at various magnifications.

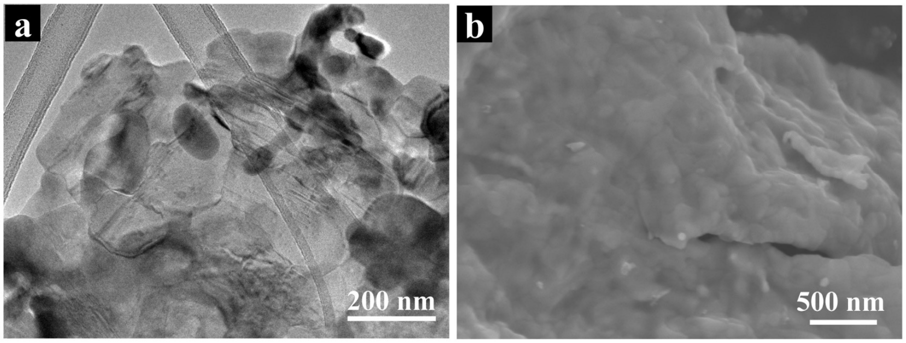

Figure S10. TEM image (a) and SEM image (b) show the MAX phase grains and grain boundaries between the Ti_2AlC nanoflakes.

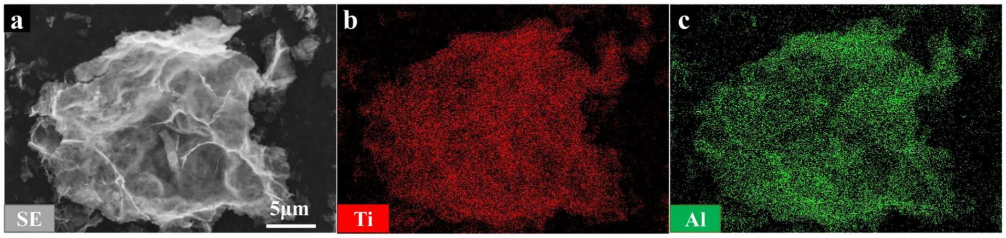

Figure S11. Distribution of Ti and Al in Ti_2AlC nanoflakes. The carbon distribution is not shown due the background noise of carbon tape used in the SEM measurement.

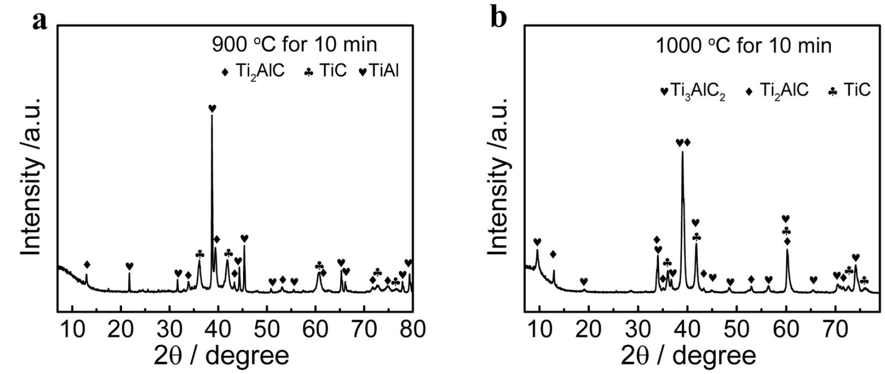

Figure S12. XRD patterns of intermediate compounds in the MAX phase synthesis after a shorter reaction time. a, Reaction products form the starting materials ratio (in mol) of Ti:Al:C= 2.3:1.3:1, held at 900 °C for 10 min. **b**, Reaction products of the starting materials ratio (in mol) of Ti:Al:C= 3:1.2:2, held at 1000 °C for 10 min. Carbon precursor used in these two sets of experiments was graphene aerogel.

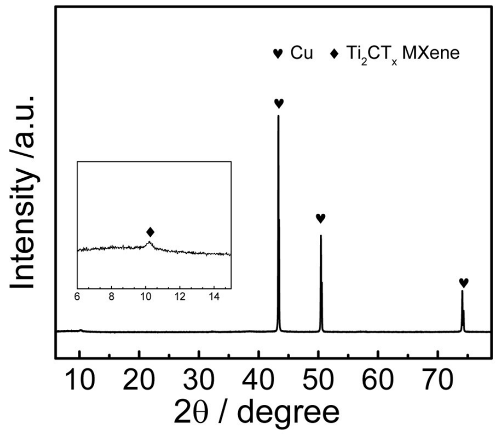

Figure S13. XRD pattern of a mixture of MXenes and Cu metal after etching at 550 °C for 0.5 h. Inset shows the low intensity peak at 2θ around 10°.

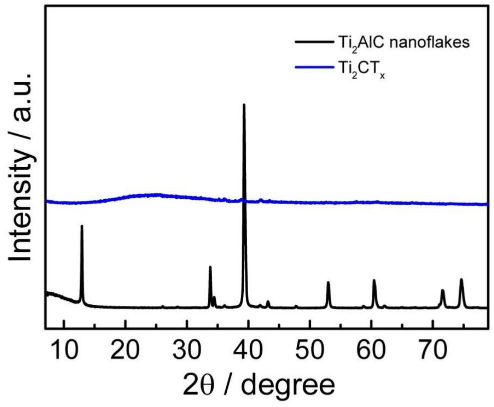

Figure S14. XRD patterns of Ti_2CT_x MXene and the corresponding Ti_2AlC nanoflakes precursor.

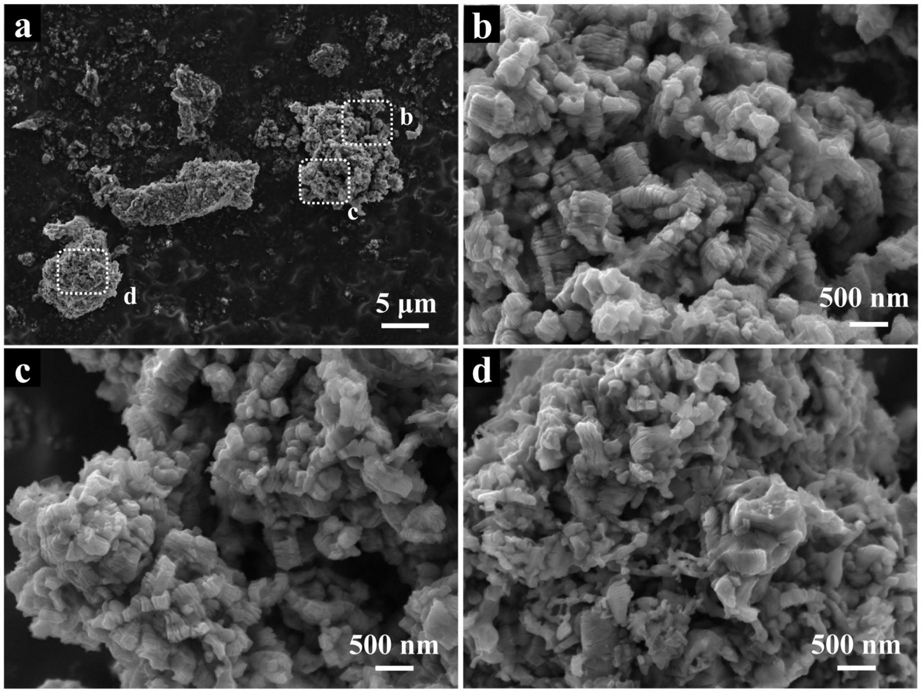

Figure S15. SEM images of Ti_2CT_x MXene prepared from Ti_2AlC nanofibers.

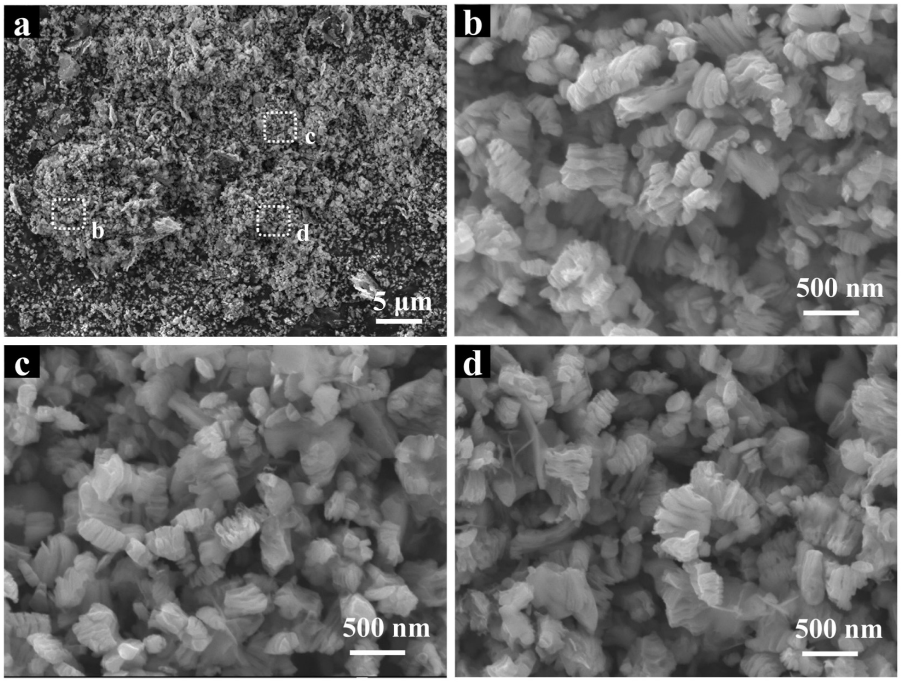

Figure S16. SEM images of $Ti_3C_2T_x$ MXene prepared from Ti_3AlC_2 nanoflakes.

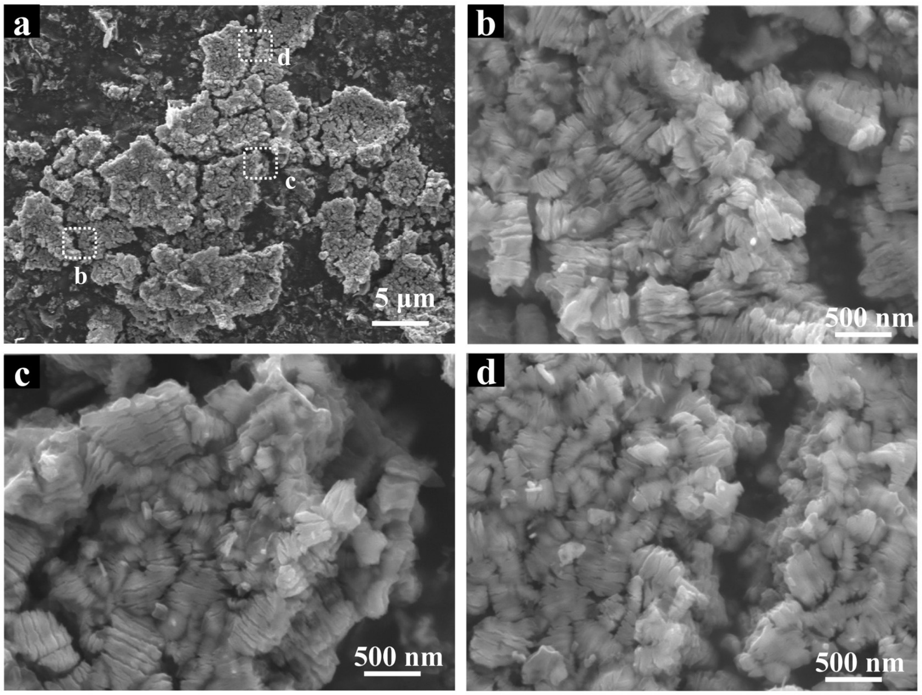

Figure S17. SEM images of Ti_2CT_x MXene prepared from Ti_2AlC nanoflakes.

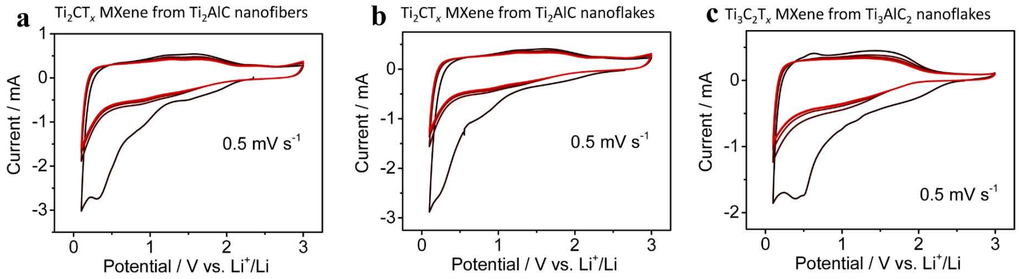

Figure S18. Cyclic voltammograms of initial five cycles at a scan rate of 0.5 mV s^{-1} of (**a**) Ti_2CT_x MXene prepared from Ti_2AlC nanofibers, (**b**) Ti_2CT_x MXene prepared from Ti_2AlC nanoflakes and (**c**) $Ti_3C_2T_x$ MXene prepared from Ti_3AlC_2 nanoflakes, respectively.

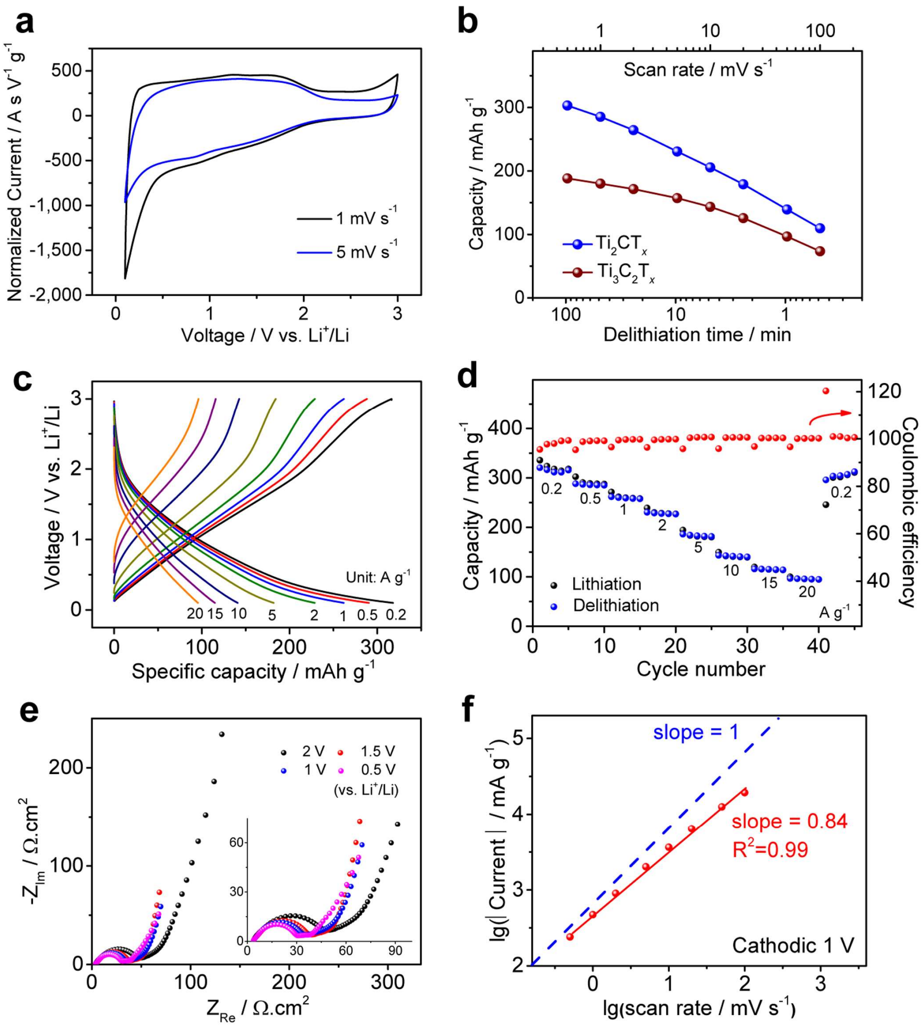

Figure S19. Li-ion storage properties of nanosized multilayered MXenes. a, Cyclic voltammetry profiles of Ti_2CT_x-NI MXene electrode at scan rates of 1 and 5 mV s^{-1}. **b,** Specific delithiation capacities of Ti_2CT_x-NI and $Ti_3C_2T_x$ MXene electrodes at various scan rates. The reported capacities represent mean values taken from three different electrochemical cells, more details in Supplementary Table S3 and S4. **c,** Voltage profiles of the Ti_2CT_x-NI MXene electrode from galvanostatic tests at various specific currents and **d,** the corresponding specific lithiation/delithiation capacities. **e,** Nyquist plots of Ti_2CT_x-NI MXene electrodes at various bias voltages versus Li^+/Li. **f,** *b*-value determination by plotting log(specific current) versus log(scan rate). The current/scan rate was taken from CV profiles in Figure S20a.

Electrochemical impedance spectroscopy (EIS) measurements were conducted at various lithiation states during the cathodic scan to further understand the electrochemical behavior of the Ti_2CT_x-NI MXene electrodes. As shown in Figure 5e, a semicircle is visible at high frequencies, corresponding to the contributions of the SEI layer and the charge transfer resistance. The diameter of this high-frequency loop decreases upon negative polarization (Figure S19e) due to decreasing charge transfer resistance upon Li intercalation, as expected for a reaction controlled by the charge transfer. In the low-frequency range, the rapid increase of the imaginary impedance indicates a pseudocapacitive Li-ion intercalation process (restricted diffusion process) instead of a diffusion-limited process (45° Warburg region).[1,2] Moreover, the charge storage kinetics of the Ti_2CT_x-NI MXene electrode was also examined by the power law[3]:

$$i = a\, v^{b} \quad (1)$$

where v is the scan rate, and i is the current response. A b-value of 0.5 means the current response is proportional to the square root of the scan rate, corresponding to a typical Warburg diffusion-controlled process which is expected for battery-like electrodes. On the other hand, a b-value of 1 means that the current response is linear with the scan rates, revealing a surface process without any diffusion limitation, typical for capacitive and pseudocapacitive electrodes.[1,4] Here, a b-value of 0.84 was found within a broad scan rate range from 0.5 to 100 mV s^{-1} for the Ti_2CT_x-NI MXene electrode (Figure S19f), suggesting that the surface reactions dominates the Li-ion intercalation/deintercalation kinetics, in good agreement with the EIS and CV results.

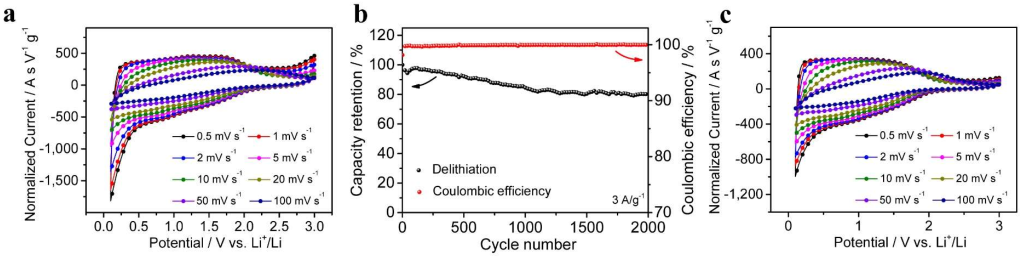

Figure S20. a, Cyclic voltammograms of a Ti_2CT_x MXene electrode prepared from Ti_2AlC nanofibers at scan rates from 0.5 to 100 mV s^{-1}. **b,** Long cycling stability of a Ti_2CT_x MXene electrode at a specific current of 3 A g^{-1}. The capacity retention of 80% was obtained after 2000 cycles. **c,** Cyclic voltammograms of a $Ti_3C_2T_x$ MXene electrode prepared from Ti_3AlC_2 nanoflakes at scan rates from 0.5 to 100 mV s^{-1}.

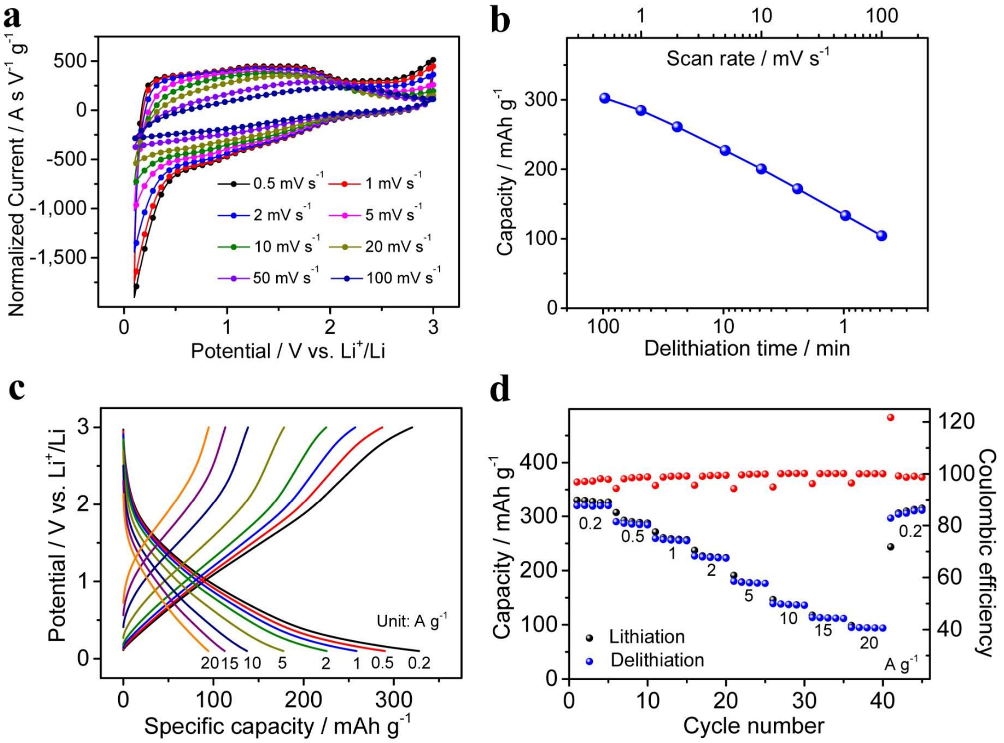

Figure S21. a, Cyclic voltammograms of a Ti_2CT_x MXene electrode prepared from Ti_2AlC nanoflakes at scan rates from 0.5 to 100 mV s^{-1}. **b**, Specific delithiation capacity at various scan rates. Note that the reported capacity is the mean value calculated from three electrochemical cells, see Table S4.

Table S3. Li^+ storage capacities of Ti_2CT_x MXene electrodes prepared from Ti_2AlC nanofibers determined from CV tests.

Scan rate, mV s^{-1}	Time, min (C-rate)	Cell 1, mAh g^{-1}	Cell 2, mAh g^{-1}	Cell 3, mAh g^{-1}	Mean value, mAh g^{-1}	Standard Deviation, mAh g^{-1}
0.5	96.7 (0.6)	301.8	298.0	307.0	302.2	4.6
1	48.3 (1.2)	284.6	281.5	287.8	284.6	3.2
2	24.2 (2.5)	262.2	256.6	264.2	261.0	4.0
5	9.7 (6.2)	227.9	222.6	230.5	227.0	4.0
10	4.8 (12.4)	201.3	196.5	203.6	200.5	3.6
20	2.4 (24.8)	174.3	168.7	172.6	171.9	2.8
50	0.97 (62.1)	137.2	130.6	132.2	133.4	3.4
100	0.48 (124.1)	108.6	102.9	101.2	104.2	3.8

Table S4. Li^+ storage capacities of Ti_2CT_x MXene electrodes prepared from Ti_2AlC nanoflakes determined from CV tests.

Scan rate, mV s^{-1}	Time, min (C-rate)	Cell 1, mAh g^{-1}	Cell 2, mAh g^{-1}	Cell 3, mAh g^{-1}	Mean value, mAh g^{-1}	Standard Deviation, mAh g^{-1}
0.5	96.7 (0.6)	299.8	307.7	301.6	303.0	4.2
1	48.3 (1.2)	281.1	292.3	282.3	285.3	6.2
2	24.2 (2.5)	262.4	269.4	261.1	264.3	4.5
5	9.7 (6.2)	228.7	234.3	229.0	230.7	3.2
10	4.8 (12.4)	204.0	208.6	204.1	205.6	2.6
20	2.4 (24.8)	178.1	181.5	177.5	179.0	2.1
50	0.97 (62.1)	138.4	141.1	138.4	139.3	1.6
100	0.48 (124.1)	109.1	111.0	109.1	109.7	1.1

Table S5. Li^+ storage capacities of $Ti_3C_2T_x$ MXene electrodes prepared from Ti_3AlC_2 nanoflakes determined from CV tests.

Scan rate, mV s^{-1}	Time, min (C-rate)	Cell 1, mAh g^{-1}	Cell 2, mAh g^{-1}	Cell 3, mAh g^{-1}	Mean value, mAh g^{-1}	Standard Deviation, mAh g^{-1}
0.5	96.7 (0.6)	188.7	189.9	186.1	188.2	2.0
1	48.3 (1.2)	178.3	182.0	179.7	180.0	1.8
2	24.2 (2.5)	169.8	173.1	171.5	171.5	1.7
5	9.7 (6.2)	155.2	157.7	158.7	157.2	1.8
10	4.8 (12.4)	141.5	142.4	146.4	143.4	2.6
20	2.4 (24.8)	124.5	122.5	130.2	125.7	4.0
50	0.97 (62.1)	97.6	90.8	101.5	96.7	5.4
100	0.48 (124.1)	76.3	67.0	77.1	73.5	5.6

Reference.

1 Mathis, T. S. *et al.* Energy Storage Data Reporting in Perspective—Guidelines for Interpreting the Performance of Electrochemical Energy Storage Systems. *Advanced Energy Materials*, 1902007 (2019).

2 Diard, J.-P., Le Gorrec, B. & Montella, C. Linear diffusion impedance. General expression and applications. *Journal of Electroanalytical Chemistry* **471**, 126-131 (1999).

3 Wang, J., Polleux, J., Lim, J. & Dunn, B. Pseudocapacitive Contributions to Electrochemical Energy Storage in TiO_2 (Anatase) Nanoparticles. *The Journal of Physical Chemistry C* **111**, 14925-14931 (2007).

4 Shao, H., Lin, Z., Xu, K., Taberna, P.-L. & Simon, P. Electrochemical study of pseudocapacitive behavior of $Ti_3C_2T_x$ MXene material in aqueous electrolytes. *Energy Storage Materials* **18**, 456-461 (2019).
